# Conformational Design and Characterisation of a Truncated Diamine Oxidase from *Arthrobacter globiformis*

**DOI:** 10.3390/ht7030021

**Published:** 2018-08-25

**Authors:** Nur Nadia Razali, Nur Hafizah Hashim, Adam Thean Chor Leow, Abu Bakar Salleh

**Affiliations:** 1 Laboratory of Molecular Biomedicine, Institute of Bioscience, Universiti Putra Malaysia, Serdang 43400, Malaysia; nadraz2001@gmail.com (N.N.R.); adamleow@upm.edu.my (A.T.C.L.); 2 Enzyme and Microbial Technology Research Center, Faculty of Biotechnology and Biomolecular Science, Universiti Putra Malaysia, Serdang 43400, Malaysia; fizahash@yahoo.com; 3 Department of Cell and Molecular Biology, Faculty of Biotechnology and Biomolecular Sciences, Universiti Putra Malaysia, Serdang 43400, Malaysia; 4 Department of Biochemistry, Faculty of Biotechnology and Biomolecular Sciences, Universiti Putra Malaysia, Serdang 43400, Malaysia

**Keywords:** diamine oxidase, protein design, homology modelling, active mini protein

## Abstract

A functional mini protein can be developed by miniaturising its size. The minimisation technique provides an excellent model system for studying native enzymes, especially in creating an alternative novel biocatalyst. Miniaturised proteins may have enhanced stability, a crucial characteristic for large-scale production and industrial applications. In this study, a huge enzyme molecule, known as diamine oxidase (DAO, comprising 700 amino acids), was selected to undergo the process. By retaining the arrangement of the original functional sites of DAO in the fourth domain, a mini DAO can be designed via homology modelling. After several downsizing processes, a final configuration of 220 amino acids displayed high binding affinity towards histamine, a short-chain substrate that was catalysed by the parental DAO. The configuration also showed enhanced affinity towards a long-chain substrate known as spermidine. The gene for the designed protein was cloned and expressed in pET102/TOPO vector and overexpressed in *E. coli* BL21 (DE3). The new mini DAO had similar temperature tolerance and versatile substrates specificity characteristics as its parental protein. An active mini-protein with these characteristics is potentially useful for several applications such as detecting biogenic amines in the biological fluids and the environment that may give rise to health issues.

## 1. Introduction

Due to the poor bio-stability and unfavourable adsorption properties of large proteins, efforts have been made to improve the affinity and selectivity of proteins [1]. A number of researchers reported numerous low-molecular weight synthetic molecules that can mimic the activity of the parental protein. Such a breakthrough may even prove useful as probes in studying molecular recognition events [2]. A mimic enzyme can be considered a part of biomimetics. This can be defined as the study of parody or acting like biological processes with non-biological compounds or living organisms such as bacteria and fungi. Biomimetic compounds are often smaller than the original compound, thus providing increased accessibility to the substrate. One of the challenges in biomimetics is to design and develop simple biomimetic systems with high catalytic efficiency and specificity that are comparable to those of the natural enzyme. Peptides and mini proteins are less accumulated in the organ so face fewer of the drug–drug interaction challenges that small molecules do. They often have minor toxicological issues emerging from xenobiotic metabolism. Certainly, they are generally effective in displaying lower toxicity compared to large compounds [3].

Presently, protein design helps in the development of new drugs and medications. Any cells, especially from plants and animals, are engineered to express antibodies and proteins used in the treatment of several illnesses and conditions. To produce high quality and yield, it is necessary for the conditions of the engineered protein one to meet optimal values, especially in the biotechnology/biosensors field. The ultimate goal in protein design is to obtain a functional protein with a desired structure. The “structural” approach and the “functional” approach are directly used to engineer the protein. The first approach is based on the de novo design of secondary motifs [4], which are assembled into tertiary structures stabilised by disulphide-bridge linkers. Meanwhile, the second approach is based on a particular function by grafting functional residues into a stable scaffold. In order to execute this process, basic information about the structure of the protein is needed. Thus, a combination of both approaches—structure-based along with the selection of the best template from the designed protein collection—can lead to the minimisation of the targeted protein [5]. Therefore, downsizing the native protein by removing unwanted regions can be a good strategy in protein mimicking.

In microorganisms, diamine oxidases (DAOs) generally have a nutritional role as the sole source of nitrogen or carbon. They have been proposed to provide a signal in wound healing in plants [6]. In mammals, they play an important role in regulating the intracellular level of amines through neurotransmitter detoxification and cell development [7]. We concluded that the interest in DAO is increasing in several fields due to its involvement in numerous physiological and metabolic pathways that are related to the biological function. The versatility of DAOs is why they were chosen as the target proteins. This knowledge is useful in understanding protein structure and function. It is hoped that this research may significantly contribute to the development of new biocatalysts in the future. Diamine oxidase was first reported in 1929 and seemed to be responsible for physiological conditions when the enzyme activity was low, especially, when there is a critical decrease of the enzyme in the metabolism of histamine [8]. Scientists are captivated by its unique characteristics in the body and brain under various conditions, especially in the evolution of a new drug [9]. Its core function is to degrade the histamine formed in tissues. DAO is a homodimer protein that consists of 60 to 105 kDa subunits [10]. As a large protein, DAO is more susceptible to destabilising agents such as heat and pH, similar to other large proteins [11]. Thus, for this study, we miniaturised the native DAO through computational modelling and protein synthesis while retaining its catalytic function. It is hoped that this research may shed light on the development of new biocatalysts in the future.

## 2. Materials and Methods

One-shot chemically competent *E. coli* BL21 (DE3) cells and the TOPO TA Cloning Kit were purchased from Invitrogen (Thermo Fisher Scientific, Waltham, MA, USA). Plasmid isolation was performed using the GeneAll DNA purification kit, from GeneAll Biotechnology (Seoul, Korea). The genomic DNA library for *A. globiformis* was purchased from DSMZ (Braunschweig, Germany). All the analyses were done in triplicate so that the variability can be estimated.

### 2.1. Homology Modelling of Diamine Oxidase

The amino acid sequence of the DAO was obtained from the UNIPROTKB/SWISSPROT database. The amino acid sequence was subjected to homology modelling using Yet Another Scientific Artificial Reality Application (YASARA) programme (Version 13.5.7) to model the 3D structure of DAO. The Position-Specific Iterated BLAST (PSI-BLAST) under the Basic Local Alignment Tool was performed against the Protein Data Bank (PDB) to search for a suitable template for protein modelling [12]. The optimised model was then subjected to a visual assessment, with respect to its geometry and energy aspects. Graphical presentations of the 3D model were also prepared using YASARA.

### 2.2. Analysing the Diamine Oxidase Domains

The targeted enzyme DAO was analysed for the existence of conserved domains. CATH was used for this analysis and supported by a web server analysis, SMART. The protein design was started by downsizing the size of DAO from N-terminal to C-terminal. Then, the structure was refined using homology modelling and structure superimposition was performed. In addition, the Ramachandran plots were executed using RAMPAGE.

### 2.3. Molecular Dynamics (MD) Simulation and Ligand Docking Analysis

MD was attained in an explicit solvent under NVT (constant number of particles, volume, and temperature) in a cubic box with periodic boundary. In two stages of equilibrium, the protein was frozen in simulation cell during the first stage and the second stage was when the equilibration of the protein was performed. The final structural conformation began when the molecular dynamic simulation was started using AMBER (AMBER03) force field. Production of MD was initiated and thereafter, the trajectory was sampled at 20 ns intervals. Concurrently, docking calculations were conducted using YASARA, which was equipped with AUTODOCK plugin. Appropriate possible ligand/substrates, such as histamine, spermidine, putrescine, spermine and cadaverine were downloaded from the PubChem as Structure Data Format (SDF) to determine the interaction between the protein and the substrate.

### 2.4. Cloning and Expression of Mini DAO

*A. globiformis* was grown in the modified agar [13,14]. This genomic DNA was isolated using DNeasy Blood and Tissue Kits (Qiagen, Germantown, MD, USA). Primers (P1: 5′ –CACCGAGCAGCTCTCGGCCGAGGAAATC- 3′ and P2: 5′- GTTGAGTTCACGCCTGTCGACGACGAGGC -3′) were used to amplify gene encoding for the mini DAO using *Pfu* DNA polymerase in a thermal cycler. The recombinant plasmids of clones that carried the gene encoding the truncated DAO were extracted and preceded to DNA sequencing analysis. The recombinant clones, harbouring recombinant plasma cells, were transformed with pET102/TOPO vector. The expression of the recombinant of mini DAO protein was conducted using *E. coli* BL21 (DE3). The mini DAO in pET102/TOPO was screened for oxidases using the plate-based oxidase activity screening method. The strains were grown at 37 °C with constant shaking (250 rpm) until the optical density of the culture at 600 nm (OD600) reached the 0.5–0.8 range. Protein expression was induced using 0.06 mM isopropyl-β-D-1-thiogalactopyranoside (IPTG) and then, 0.05 µM copper sulphate (CuSO_4_) was added. Thereafter, the culture was further cultivated at a lowered temperature of 25 °C on a rotary shaker for 18 h. The cell was harvested at 10,000× g for 30 min at 4 °C and the pellet was re-suspended in 5 mL of 50 mM phosphate buffer (pH 7.4). Cells were disrupted by sonication in ice (5 × 1 min, output control 4, duty cycler 40%). This was followed by centrifugation at 12,000× g at 4 °C for 20 min to remove cell debris and insoluble proteins. The expressed protein in the soluble fraction was subjected to protein purification. Next, it was analysed using native polyacrylamide gel electrophoresis (PAGE) and separated using SDS-PAGE. Then, the cells were transferred onto nitrocellulose membrane for immunoblotting analysis.

### 2.5. Purification of Mini DAO

The recombinant mini DAO was purified using a Nickel-Sepharose HP column. The recombinant fraction containing soluble protein was applied to a 10 mL Nickel-Sepharose column (1.6 × 20 cm). This column had been previously equilibrated, with 50 mM potassium phosphate buffer (pH 7.4) that contained 0.5 M of sodium chloride, at the flow rate of 1 mL/min. The column-bound enzyme was washed with 50 mM potassium phosphate buffer (pH 7.4), containing 500 mM imidazole and 0.5 M sodium chloride. Elution was conducted with a linear gradient of imidazole (0–500 mM) in 50 mM potassium phosphate buffer (pH 7.4). The purified enzyme was subjected to peroxidase activity determination [15].

### 2.6. Measurement of Mini DAO Activity and Stability

To study substrate specificity, DAO reactions against various biogenic amines were determined enzymatically by monitoring the release of hydrogen peroxidase at 440 nm by using the DAO standard assay. The chromogenic solution was prepared by mixing 2.5 mL of sodium phosphate buffer (100 mM, pH 7.2), 0.2 mL of 75 mM substrate, 0.1 mL of 2000 pyrogallol/unit of horseradish peroxidase (HRPO) and 0.1 mL of 16 mM o-dianisidine (ODA). The mixture was equilibrated at 37 °C for 10 min, then 0.1 mL of crude enzyme was added into it. The absorbance was recorded at 440 nm. The level of activity produced by DAO was measured by determining the amount of hydrogen peroxide (H_2_O_2_) released based on the standard curve of H_2_O_2_. One unit of diamine oxidase activity is defined as the oxidation released 1 μmole of H_2_O_2_ per min at pH 7.2 at 37 °C [16]. The oxidase activity was measured at temperatures between 20 to 50 °C after the assay mixture had been equilibrated for 20 min at the assay temperature. The optimum pH for histaminase oxidase was determined at different pH values at 40 °C. The following buffers were used: 50 mM acetate buffer (pH 4–6), 50 mM phosphate buffer (pH 6–8), 50 mM Tris-HCl buffer (pH 8 and 9), 50 mM glycine-NaOH buffer (pH 9–11) and 50 mM Na_2_HPO_4_-NaOH buffer (pH 11–12). The enzyme activity was measured using the standard DAO assay. For the determination of kinetic parameter, mini DAO activity was measured at different substrate concentrations at 40 °C using the DAO standard assay.

## 3. Results and Discussion

### 3.1. Molecular Analysis of Mini Diamine Oxidase from A. globiformis

In this study, the aim was to generate a mini functional protein from the naturally-appearing large DAO structure. Protein design is important to test the limits of completeness of the structure before modifying or reconstructing proteins and to allow the creation of novel enzymes for biotechnological applications [17]. The strong relationship between sequence analysis and protein structural study was due to significant sequence conservation, functional similarity, and local structural resemblance. The amino acid sequence of a DAO was obtained from the UNIPROTKB/SWISSPROT database, using the protein knowledge database with the entry name diamine oxidase. There were 55 entries for DAOs from various types of eukaryotes and prokaryotes in the UniProtKB/Swiss-Prot database. Surprisingly, only nine out of 180 nucleotide sequences were obtained with complete protein sequence, and with no alterations or dismissal. The entry number Q59118 (histamine oxidase from *Arthrobacter globiformis*) was chosen to be the representative for the DAO protein [14].

### 3.2. Homology Modelling of Mini Diamine Oxidase

**Figure 1:**
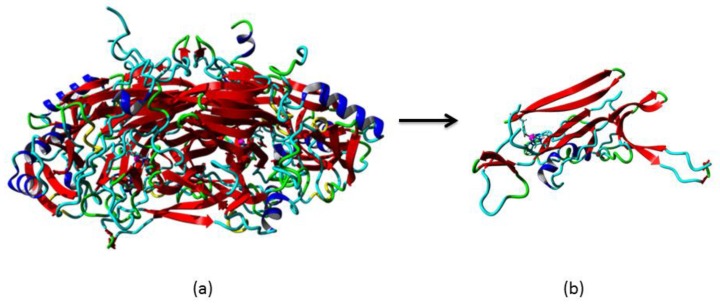
The structure of (**a**) native DAO and (**b**) mini DAO after minimisation. Illustration was taken by YASARA^TM^.

**Figure 2:**
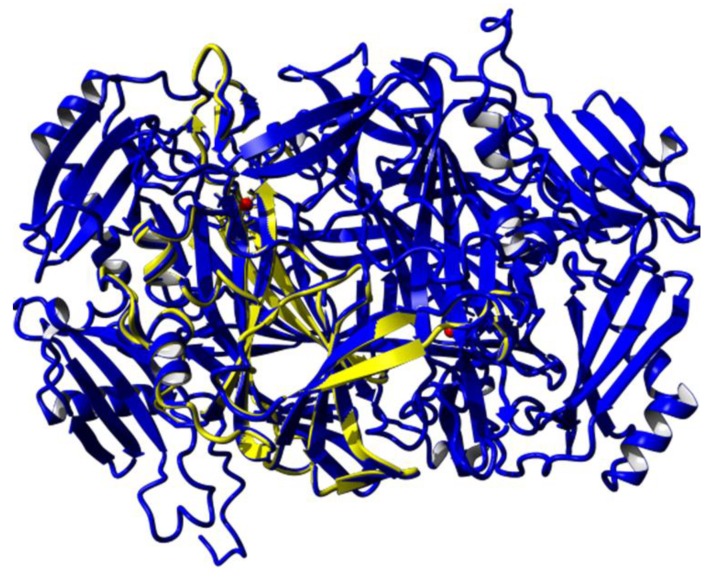
Superposition of Cα traces of mini DAO and template by minimisation approach. The colour indicates mini DAO (yellow), parental DAO (blue) or copper ion (red). The RMSD value of the superposed structure was 0.328 Ǻ.

**Figure 3:**
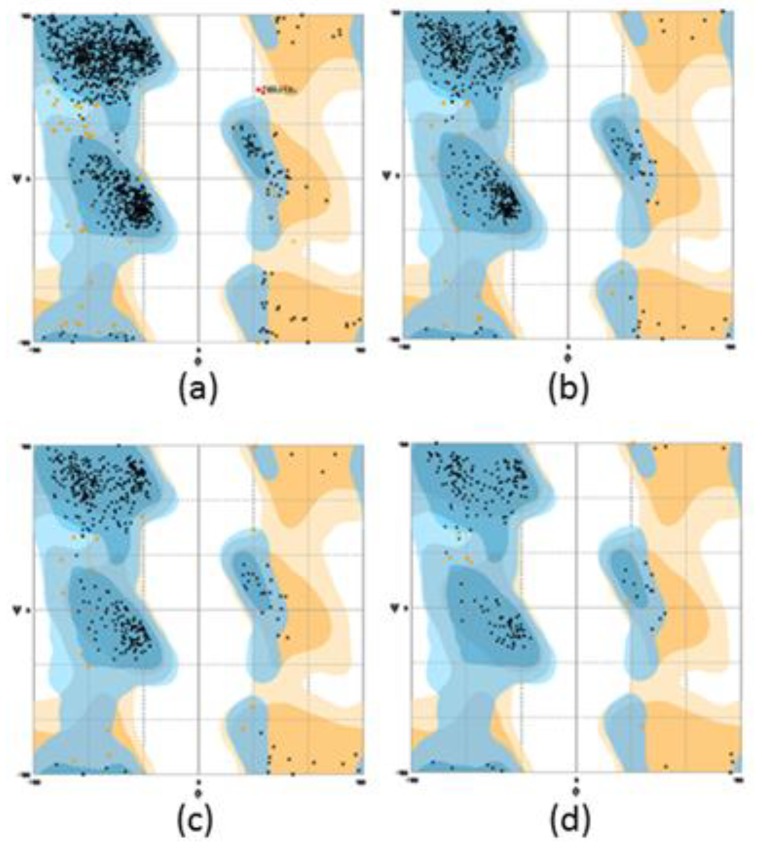
The Ramachandran plot for DAO and its domain. (**a**) AGAO, (**b**) native DAO, (**c**) domain 4 and (**d**) mini DAO.

Homology modelling is one of the most common structure prediction methods for three-dimensional protein structures, aside from NMR and X-ray crystallography [18]. PSI-Blast was performed for each protein sequence against the PDB database in order to identify the template structure [17]. A structural model must exceed more than 35% sequence identity to the known 3D template [18]. Thus, the CLUSTALW revealed that amine oxidase from *A. globiformis*, AGAO (Protein Data Bank code: 1IVU), is a suitable template to build the mini DAO with sequence match of 61% homology to DAO [20]. In 2009, AGAO was reported to have a narrow channel similar to human diamine oxidase, *h*DAO [6]. In fact, the catalytic area of tetra-peptide sequence, (Asn-Tyr-Asp-Tyr) was also conserved in the structure [7]. With the percentage of higher than 60% similarity, the AGAO model was engineered using YASARA software. The open reading frame contained 2052 base pairs (bp) or nucleotide-encoded the protein comprising 684 amino acids. Sequence analysis using NCBI-Blast showed that the reduced amino acid sequence contained a highly conserved amine oxidase family domain, with zero E-value, which described the absence of background noise [21]. Subsequently, the model was subjected to a Kyte–Doolittle plot, whereby the protein was plotted below 0, which indicated that the side-chain packing had excellent hydrophobic regions that were exposed on the surface of the protein [14]. All of these results signified that the 3D structure of the mini DAO was trustworthy. The DAO protein was a homodimer protein (monomers A and B) [6]. Monomer A was submitted to conserved domain analysis. Sequence analysis using NCBI conserved domain database (CDD) found that this monomer contained three conserved domains, namely, Domain 2 (D02), Domain 3 (D03), and Domain 4 (D04). The catalytic region was located in D04 and exhibited a high sequence similarity to amine oxidase superfamily [23]. This analysis was in agreement with the CATH Domain and SMART analyses, which showed the presence of the functional domain of DAO [24]. With the aim of miniaturising the protein while maintaining its active site, D04 was subjected to the next step. D04 was cropped from the N-terminal, starting from the first amino acid residue up to 158 amino acids. Meanwhile, at the C-terminal, 33 amino acid residues were cropped. The splicing process was resumed until the structure collapsed in silico. The final conformation was obtained with a structure that had 220 amino acid residues (Figure 1). The model of the mini DAO was considered reliable and supported by superimposition of the Cα result [25]. Superimposition of the polypeptide backbone of both mini DAO model and the template (PDB: 1IVU) gave forth an RMSD value of 0.328 Å over 218 aligned residues with 98% sequence identity (Figure 2) [26]. The minimisation process was considered to be successful because the mini DAO model was able to retain structural similarity and sequence identity. It shared similar interactions towards its ligand and substrates. The native protein and the model of mini DAO were also subjected to structural quality assessment and the model was validated for psi and phi torsion positions using the Ramachandran Plot by RAMPAGE [23]. The analysis revealed that 98% and 2% of the residues of the mini DAO model were placed in the favoured and allowed regions, respectively (Table 1 and Figure 3). Additionally, the value of the goodness (G) factor from PROCHECK was obtained in the acceptable range (−0.424), thus, indicating the reliability of the model. The validation result showed that the stereochemistry of this model was reasonably accurate. Addition, the GRAVY index of −0.424 proved that the mini DAO was hydrophilic and soluble compared to the parental protein, with −0.212. Simultaneously, the Z-score for the native DAO was −9.11, and −7.51 and −3.75 were obtained for the D04 and mini DAO, respectively, according to ProSA. This analysis was in agreement with a secondary structure analysis, with i-Tasser. The values suggested similarities between the template and the modelled structure, while the negative values indicated that the structure was stable. The comparable Ramachandran plot characteristics, RMSD values, and Z-scores, therefore, were considered reliable for subsequent in silico studies [27]. The amino acid sequence has a predicted isoelectric point (PI) of 5.18 and molecular weight of 75,000 Da for native DAO, whereas for the mini DAO the PI was 5.23, with the molecular weight of 24,000 Da. Moreover, the instability index prediction for the native and mini DAO was 29.10 and 28.33, respectively, which were lower than 40. These values indicated that the protein was stable, even though some amino acid residues had been removed. Chopping the huge DAO may also affect its solubility in water. This analysis was in agreement with a secondary structure analysis, Protparam. Meanwhile, according to the PROSO evaluator, the value must exceed 0.500 to be classified as a soluble protein [28]. The PROSO value for the native DAO was 0.599, with 58.4% chance of solubility when overexpressed in *E. coli*. Additionally, D04 consisted of 411 amino acid residues with 64.2% predicted solubility at 0.699 of the PROSO value. The PROSO value for the mini DAO was 0.549 and there was a 60% chance of solubility when overexpressed in *E. coli*. These solubility values could later be indicators in the recombinant protein expression. Their solubility model was based on two parameters; (i) the average charge determined by the relative numbers of Asp, Glu, Lys, and Arg residues, and (ii) the content of turn-forming residues (Asn, Gly, Pro, and Ser) [28]. Apart from that, the software can also perform discriminative analyses, such as checking the charge average, turn forming residue fraction, cysteine fraction, proline fraction, hydrophobicity and the total number of residues [29].

### 3.3. Molecular Dynamic Simulation and Protein Docking Analysis

**Figure 4:**
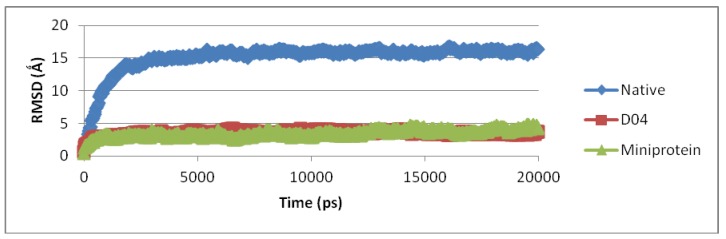
Trajectories for RMSD for native DAO, D04, D03 and mini DAO for 20 ns. The protein was indicated as in the legend.

Given its morphological complexity, the model was submitted to a molecular dynamics (MD) simulation in water for over 20 s at 300 K to determine its conformational viability. The results, as summarised in Figure 4, had established that the mini DAO had a reasonably well-preserved fold in water, with RMSD values ranging between 2.0 to 2.3 Å [30]. The mini DAO structure had actually maintained its properties as D04. The RMSD values could be compared even though the sizes were different. Therefore, the RMSD values were maintained along trajectories that were considered stable for both native and mini DAO. The fluctuation in the RMSD values was mainly due to the loss of local structure. Reassured of the possible hydrophobic effect of assisted fold in water, the protein was synthesised and experimentally characterised.

Commonly, docking predicts the favoured orientation based on the binding energy of one ligand to a receptor. The knowledge of favoured orientation, in turn, can be used to predict the binding affinity between two molecules using a scoring function. The structural pockets and cavities often determine the binding and active sites of proteins. The catalytic site of DAO was obtained from the information available in the literature [31]. The local docking was performed when the active site/binding pockets of the native and mini DAOs were used to make the binding more specific. Based on the docking results shown in Table 2, various carbon chain length substrates were used to interact with the mini DAO, which showed similar interactions with the short-chain substrates the native protein [32]. Surprisingly, the mini DAO also had good interactions with long-chain substrates. The positive and high value of binding energy indicated a possible interaction between the protein and the ligand/substrate in certain conformations. The docking analysis with a series of ligands revealed that there were important residues involved in the catalytic activity. This proved that the conformation of the binding pocket was maintained, even though the size was one-third that of the parental DAO [33]. During the catalysis process, the structure of the apo-form of the mini DAO revealed a phenolic hydroxyl group of the precursor tyrosine pointing towards the vacant metal binding sites, comprising three conserved histidine residues. The N3 of imidazole that was located opposite the TPQ formed a hydrogen bond with the aspartic acid residue. Another imidazole side in the substrate channel formed a hydrogen bond to another tyrosine and made a water-mediated contact with the main chain nitrogen of threonine [34]. In the productive docking geometry, the distance between the protein and the ligands should not exceed 5 Å [32].

### 3.4. Expressing of Recombinant Mini DAO in *E. coli* Cells

**Figure 5:**
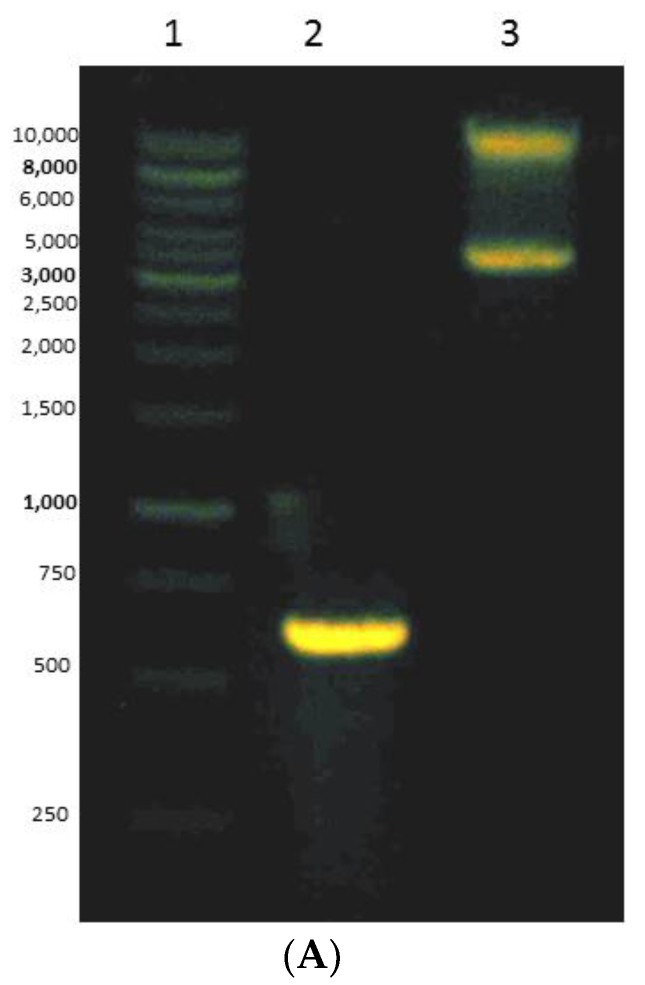

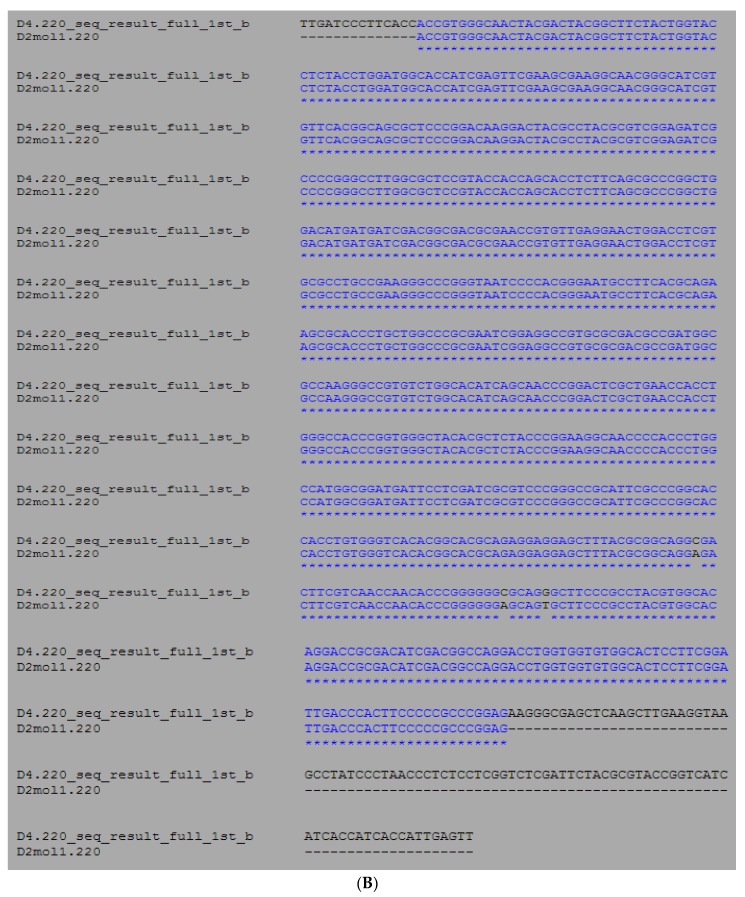
(**A**) Gel electrophoresis of recombinant plasmid verifies the positive transformant. The recombinant was electrophoresed on 1% (*w*/*v*) agarose gel. Lane 1: Marker GeneRule 1kb DNA; Lane 2: gene of interest; Lane 3: extraction plasmid. (**B**) Sequence alignment of mini DAO sequence after sequencing and designed targeted mini DAO generated in silico in Biology Workbench 2.0 (http://workbench.sdsc.edu/).

A gene encoding for the mini DAO was cloned into pET102/TOPO vector downstream of the His-tag under the regulation of T7 lac operator-promoter and was heterogeneously overexpressed in *E. coli* BL21 (DE3) (Figure 5A). The recombinant was electrophoresed at 1% (*w*/*v*) agarose gel). The cell was grown in the presence of IPTG and CuSO_4_ [35]. The plasmid extraction was subjected to sequencing service to verify the similarity between the targeted mini DAO and the template (Figure 5B). There were only two points of mutation at the alanine and glycine amino acid residue, where only the nucleotides had changed, but the amino acid had maintained its original state. Optimisation expression revealed that a higher expression level could be achieved when the culture was induced with 0.02 mM IPTG at 25 °C for 24 h.

### 3.5. Purification of Fusion Mini DAO

**Figure 6:**
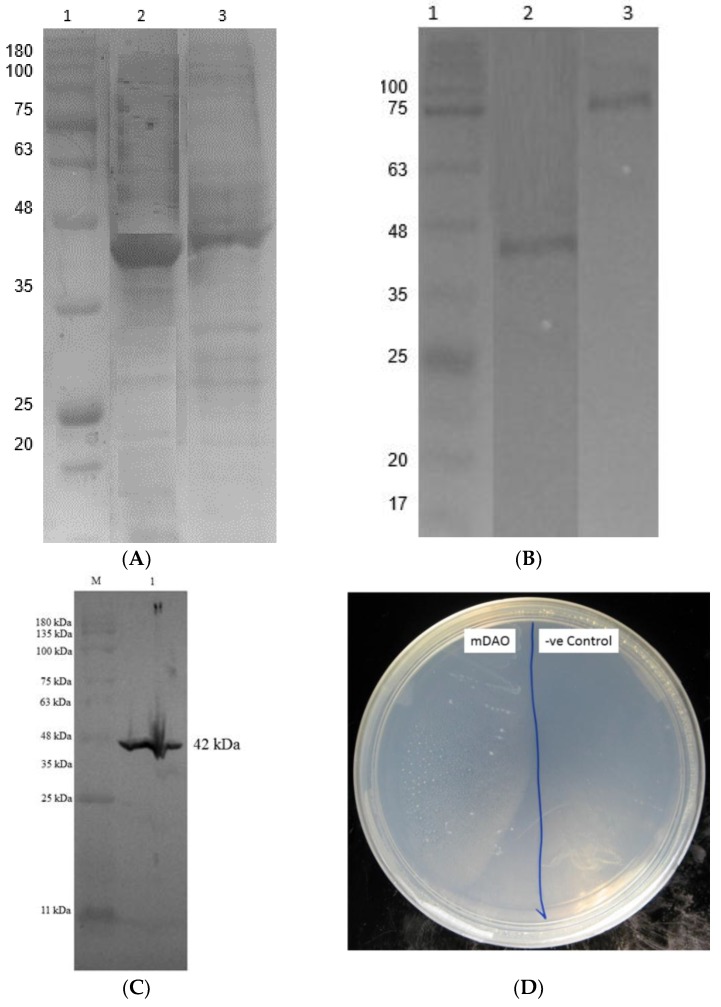
(**A**) SDS-page (12%) of purified recombinant fusion mini DAO through affinity chromatography with the crude enzyme. Lane 1: Blue-eye Prestain Protein Marker (Genedirect, USA); Lane 3: Crude enzyme of fusion mini DAO; Lane 2: purified fusion mini DAO. Expected size was 42.2 kDa. (**B**) Verification of recombinant mini DAO by native PAGE. Lane 1: Prestained molecular weight marker, Lane 2: Fusion His-tag mini DAO at 42.2 kDa and Lane 3: native DAO, size 92 kDa. (**C**) Verification of recombinant mini DAO by immunoblotting method. M: prestained molecular weight marker, Lane 1: Fusion His-tag mini DAO on nitrocellulose membrane at 42.2 kDa. (**D**) Plate screening of histaminase and the negative control with presence of histamine as the substrate.

The fusion mini DAO was purified from the cell -free extract of AGAO to homogeneity using a single-step affinity chromatography using Ni^2+^ Sepharose resin (Table 3). The enzyme was purified approximately 2-fold over the cell-free extract, with a yield of 88% and the specific activity of 42 U/mg. The purified enzyme was observed by the brownish pink colouring of the solution with the absorption maximum at about 440 nm. To check the purity of the purified mini DAO, the final enzyme preparation was subjected to native-PAGE and SDS-PAGE. The enzyme showed a single band, as observed on native-PAGE. The purified enzyme was homogeneous on both native-PAGE and SDS-PAGE. The mini DAO was shown to be in the monomer form. The molecular mass of the purified fusion mini DAO was estimated to be approximately 42.2 kDa by SDS-PAGE, native-PAGE, and Western blotting (Figure 6A–C). Screening plate method was used in confirming the functioning mini DAO’s activity. *E. coli* strains that harboured the recombinant plasmid and empty vector in *E. coli* BL21 (DE) were checked for the presence of histamine as a substrate. The recombinant *E. coli* carrying the gene of the mini DAO showed cell growth, while no growth was observed for the negative control (Figure 6D) [36].

### 3.6. Characterisation of Mini DAO

**Figure 7:**
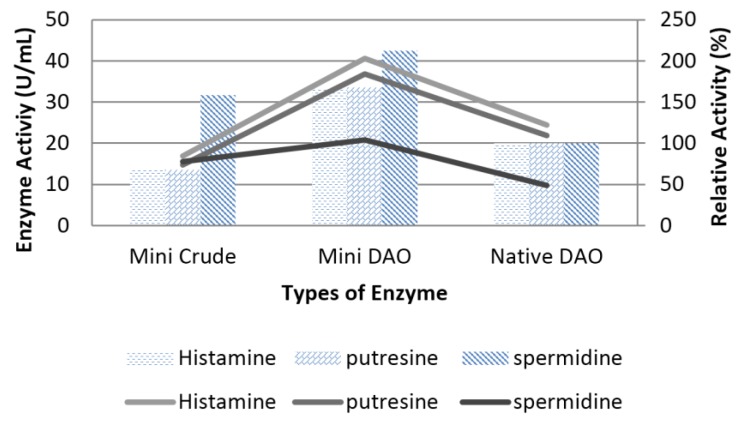
Substrates specificity on different carbon chain length of biogenic amine. The substrates used were histamine (C5, imidazole ring), putrescine (C4) and spermidine (C7).

**Figure 8:**
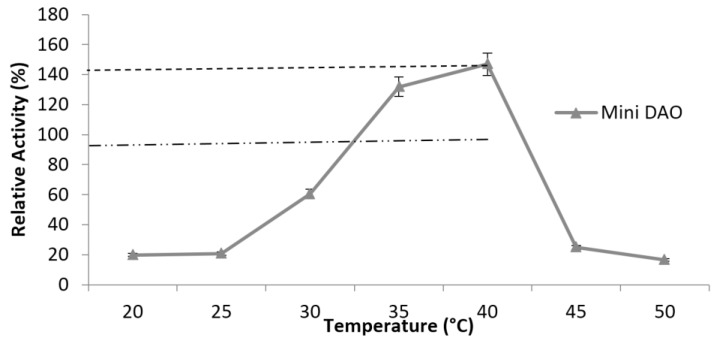
The effect of optimisation of temperature on mini DAO activity and native DAO. The activity was found by operating a histamine (C5) assay in the temperature range 20 to 50 °C in 50 mM phosphate buffer, pH 7.2.

**Figure 9:**
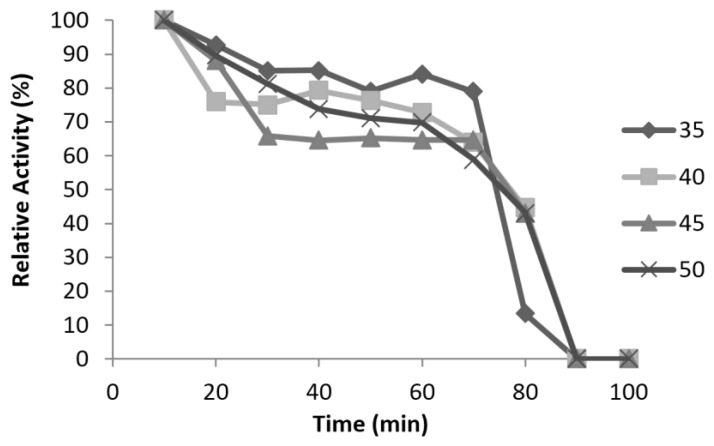
The effect of temperature on mini DAO stability. The enzyme was pre-incubated at 35 °C, 40 °C, 45 °C and 50 °C.

**Figure 10:**
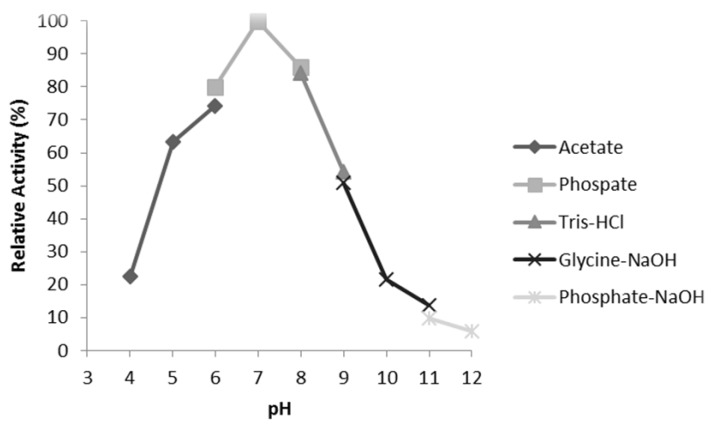
The effect of pH on mini DAO. The activity was determined at 40 °C along the physiological pH from 4.0 to 12.0 using histamine (C5) as a substrate. The buffers used were: 50 mM acetate buffer (pH 4–6), phosphate buffer (pH 6–8), tris-HCl (pH 8 and 9), glycine-NaOH (pH 9-11) and phosphate-NaOH (pH 11 and 12).

**Figure 11:**
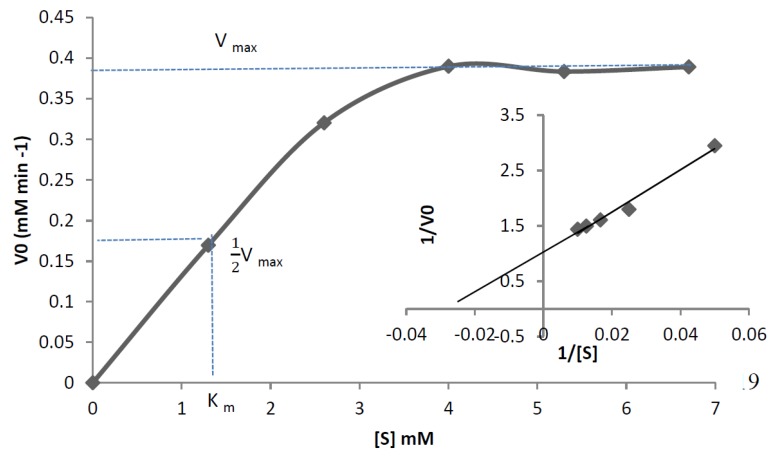
Initial velocity versus substrate concentration plot for enzyme-catalysed reaction. The curve complies with the Michealis–Menten equation when *R*^2^ = 0.95. The K_m_ and V_max_ values were determined using standard nonlinear regression techniques. (Insert) the corresponding Lineweaver–Burk plot for mini DAO catalysed reaction.

In addition, the purified recombinant mini protein was used to catalyse various kinds of substrates at 40 °C and at pH 7, especially with spermidine, while maintaining the interaction with histamine like the native DAO (Figure 7). The results demonstrated that the mini DAO had a strong catalytic ability against both short-chain and long-chain substrates (C5 and C8). However, the substrate specificity of the mini DAO significantly differed from the native DAO. The mini DAO showed optimal activity towards a long-chain substrate (spermidine), in which the relative activity was 120% higher than the native DAO. On the other hand, mini DAO also showed high activity (170% relative to histamine and putrescine) with short-chain substrates as compared to the native DAO. The substrate preference of the mini DAO was similar to the parental DAO, with higher efficiency towards histamine and putrescine, as expected [37]. This indicated that the recombinant mini DAO could function as well as the parental DAO, even though it was smaller. To determine the effect of temperature, the enzyme in 0.1 M phosphate buffer (pH 7.2) was incubated at different ranges of temperature (20–50 °C) for 10 min. The results showed that the mini DAO retained its activity during heat treatment at 40 °C for 10 min, and no activity was detected at higher than 50 °C (Figure 8). Nevertheless, the optimum temperature of the mini DAO was higher than the native DAO, which was functionally active only up to 37 °C [38]. The recombinant mini DAO, on the other hand, was active even at low temperatures. However, when the temperature reached 45 °C, the enzyme began to lose its catalytic function. The native enzyme completely lost its activity under the same conditions. Apparently, the weak hydrogen bond between the enzyme and the substrate was interrupted at this point. Additionally, hydrophobicity and other secondary interactions of the enzyme might reduce the conformational flexibility at higher temperatures. This could also affect the enzyme’s ability to recognise and attain a proper conformation in order to keep its reactivity [34]. As claimed by a previous report, the enzyme from *Aspergillus niger* AKU3302 showed its thermo-stability below 35 °C, at pH 7.0 for 10 min [39]. Thus, it was proven that the mini DAO was more heat-resistant compared to the native DAO and the DAO from *A. niger*. As for the stability findings, the mini enzyme retained 100% of its initial activity at 40 °C up to 120 min and became inactive at 45 °C, after 80 min of treatment (Figure 9). The half-life of the mini DAO was 80 min at 45 °C, which was slightly higher than the reported native DAO, with a half-life of 50 min at 35 °C [38]. The effect of pH on the activity of the purified mini DAO was determined in various buffers. The optimum pH of the mini DAO was found to be similar to the native DAO. The higher activity detected in this range had proven that the mini DAO was a good potential alkaline enzyme. This was significant for the protonation of the cleft residues in the catalytic area because of high acid or basic environment, which might have caused the activity and the coordination of the structure to decline or change. At acidic buffer, pH 4–5, low activity was detected and the activity slightly declined when the pH was increased up to 12 (Figure 10). As for the K_m_ measurement, the purified recombinant mini DAO was incubated with various concentrations of histamine. The final concentration was between 20 and 100 mM. The K_m_ value of the mini DAO, estimated from the Michaelis–Menten plot and the corresponding Lineweaver–Burk plot (Figure 11), was 1.3 mM, with histamine as the substrate. The K_m_ for a native DAO was found to be 0.274 mM with the same substrate. Therefore, the wild-type oxidase has a better affinity and better catalytic activity compared to the mini DAO. A lower K_m_ value would result in a faster maximum velocity attained with a greater affinity of the substrate to the enzyme. There would have been a decrease in substrate concentration to give the particular velocity [40].Thus, it was concluded that the mini-protein was less efficient with a short-chain substrate compared to native DAO.

## 4. Conclusions

The miniature diamine oxidase was successfully developed using Computer Design (homology modelling) and molecular methods (cloning and protein expression), which elicited a novel enzymatic strategy for designing small proteins that can mimic parental DAO. Accordingly, the mini DAO was more reliable in short MD simulation and had better interactions with histamine (C5) and spermidine (C7). For protein expression, the relative molecular weight of the native DAO was 92 kDa, while the mini DAO was 42.2 kDa, which was 32% smaller than the original size. At 40 °C, and pH 7, the mini DAO was highly activated and managed to remain stable at this condition compared to the native protein, which was stable only up to 37 °C. For protein purification, the mini DAO was successfully purified by 2-fold, with 88% recovery. The mini DAO was able to react with histamine (C5) and spermidine (C7), which was in alignment with the computational analysis results. It was also more able to resist heat than the native DAO. In conclusion, the fruitful information gathered from this study concludes that the mini DAO has improved stability and versatility, even though it is smaller, while uniquely showing affinity towards both short-chain and long-chain substrates. The mini DAO has great potential in many industrial applications, especially in the fishery industry. The summary of the comparison between mini DAO and the native DAO was shown in Table 4.

## Figures and Tables

**Table 1:** Ramachandran values for the predicted DAO and its conserved domain in favoured, allowed and outlier regions using Rampage Ramachandran Plot Server.

Protein	Number of Residues in Favoured Region (98.0% Expected)	Number of Residues in Allowed Region (~2.0% Expected)	Number of Residues in Outlier Region
HDAO	97.7	2.3	0
AGAO	96.4	3.5	0
D4	96.7	3.3	0
Mini DAO	98.0	2.0	1

**Table 2:** The binding energy (kJ/mol) of the protein–ligand interaction with various substrates.

Substrates	Natives	D04	D03	D02	Mini DAO
Histamine	8.92	7.45	4.11	3.66	8.13
Putresine	7.04	6.42	4.82	4.04	6.92
Cadaverine	6.04	5.56	6.39	3.92	5.99
Spermidine	5.92	5.46	4.52	3.64	5.99
Spermine	5.16	4.60	4.36	3.50	5.13

**Table 3:** Purification table of intracellular expressed fusion mini DAO.

Purification Step	Total Protein (mg)	Total Activity (U)	Specific Activity (U/mg)	Fold	Yield (%)
Crude	19.613	438.929	22.380	1	100
Ni^2+^-Sepharose	11.872	498.265	41.970	1.875	88

**Table 4:** Summary of characterisation of native and mini DAO.

Properties	Native DAO	Mini DAO
Molecular weight (SDS-PAGE)	~74 kDa	~24 kDa (32% smaller)
Temperature	35 °C	40 °C
pH	7	7
Substrate specificity	Histamine (C5)	Histamine (C5), Spermidine (C7)
Half-life	50 min at 37 °C, no activity at 50 °C	80 min at 50 °C
K_m_ Value	0.274 mM	1.3 mM

## Abbreviations

DAO	Diamine Oxidase
DNA	Deoxyribonucleic acid
YASARA	Yet Another Scientific Artificial Reality Application
PDB	Protein Data Bank
3D	Three-dimensional
MD	Molecular dynamics
NVT	(constant number of particles, volume, and temperature
SDF	Structure Data Format
OD	Optical Density
IPTG	isopropyl-β-d-1-thiogalactopyranoside
PAGE	polyacrylamide gel electrophoresis
SDS-PAGE	sodium dodecyl sulphate polyacrylamide gel electrophoresis
ODA	Dianisidine
NMR	Nuclear magnetic resonance
AGAO	*Arhrobacter globiformis*
hDAO	Human diamine oxidase
CDD	Conserved domain database
RMSD	Root-Mean-Square deviation
